# Genome-Wide Identification of *PSK* Gene Family and Effects of Abscisic Acid (ABA) in Regulating Antioxidant Activity and ROS Signaling Under Drought Stress in *Brassica napus*

**DOI:** 10.3390/ijms27021064

**Published:** 2026-01-21

**Authors:** Xiaojing Zhang, Zeeshan Ghulam Nabi Ghishkori, Iqbal Hussain, Muhammad Haseeb Javaid, Guangqi Zhu, Jiabao Huang, Rana Muhammad Amir Gulzar

**Affiliations:** 1 College of Biology, Hunan University, Changsha 410082, China; zxj926@hnu.edu.cn; 2 Institute of Vegetable Science, Zhejiang University, Hangzhou 310058, China; iqbal@zju.edu.cn (I.H.); haseeb.javaid@zju.edu.cn (M.H.J.); 12516082@zju.edu.cn (G.Z.); 3 Key Laboratory of Biology of Crop Pathogens and Insects of Zhejiang Province, Institute of Biotechnology, College of Agriculture and Biotechnology, Zhejiang University, Hangzhou 310058, China; 12116150@zju.edu.cn

**Keywords:** phytosulfokine, *Brassica napus*, genome-wide, expression profile, drought, abscisic acid

## Abstract

Phytosulfokine (PSK) is a tyrosine-sulfated pentapeptide found throughout the plant kingdom, playing key roles in plant growth, development, and responses to biotic and abiotic stresses. However, there is still a lack of a comprehensive analysis of the *BnPSK* gene family in *Brassica napus*. In this study, we conducted a genome-wide identification and characterized 19 *BnPSK* genes in oil seed plants, which are unevenly distributed across both sub-genomes (A and C). BnPSK proteins ranged from 77 to 99 amino acids (BnPSK3c and BnPSK3d) in length, all belonging to the PSK-α type and containing conserved PSK domains. Synteny analysis revealed that the expansion of the *BnPSK* gene family is primarily attributed to whole genome duplication, with homology to *Arabidopsis thaliana PSK* genes. A promoter region analysis identified *cis*-acting elements related to hormone and stress responses. An expression profile analysis showed that *BnPSK* genes are highly expressed in roots, leaves, petals, and pollens and are induced by both abiotic stresses and phytohormone application. Furthermore, RT-qPCR assay demonstrated that the expression levels of *BnPSK4c*, *BnPSK5a*, and *BnPSK5b* were significantly enhanced under drought stress (3~5-fold) both in plant roots and leaves following ABA application. Lastly, the application of ABA induced antioxidant activity including SOD, POD, CAT and APX (2~5-fold) and their corresponding genes (3~5-fold), and altered the ROS-signaling in rapeseed plants; also, strong evidence of mitigating drought stress was present. These findings establish a basis for further research into the role of the *BnPSK* gene family in oilseed plant tolerance against drought stress and underlying molecular mechanisms, offering valuable perspectives for developing novel peptides.

## 1. Introduction

Polypeptides, ranging from a few to several hundred amino acids, serve as fundamental signaling molecules that regulate plant growth, development, and adaptation to abiotic and biotic stresses [1,2]. These bioactive peptides primarily facilitate intercellular communication through specific ligand–receptor interactions [1]. Genome-wide studies have identified a diverse family of sulfated peptides, with the biological functions of several members being increasingly characterized [3,4,5]. Among them, phytosulfokine (PSK) has emerged as a pivotal sulfated peptide hormone involved in a wide range of physiological, developmental, and immune processes [6,7]. Initially isolated from *Asparagus officinalis* [7], PSK peptides are derived from precursor proteins of approximately 75–123 amino acids and are evolutionarily conserved across plant species [8]. Based on conserved tyrosine-sulfated core motifs, the PSK family is classified into four major types: PSK-α (YSO_3_HIYSO_3_HTQ), PSK-γ (YSO_3_HVYSO_3_HTQ), PSK-δ (YSO_3_- HIYSO_3_HTN), and PSK-ε (YSO_3_HVYSO_3_HTN) [7,9].

Accumulating evidence reveals that PSK signaling plays complex, context-dependent roles in plant immunity. For instance, silencing PSK pathway components increases tomato susceptibility to *Botrytis cinerea*, while PSK-induced immunity requires its receptor PSKR1 [9]. In contrast, PSKR1-mediated signaling can attenuate the defense against *Pseudomonas syringae* in *Arabidopsis thaliana*, and exogenous PSK application promotes growth [10]. PSK also enhances susceptibility to downy mildew in *A. thaliana* [11]. Nevertheless, the overexpression of *AtPSK2*, *AtPSK4*, or *AtPSKR1* markedly enhances pathogen resistance, highlighting the multifaceted nature of *PSK* in plant defense [12]. Beyond biotic stresses, *PSK* participates in abiotic stress responses, such as chilling tolerance in loquat fruit [13] and heat, drought, and salt tolerance in rice [14]. Additionally, PSK regulates various developmental programs, including stimulating mitotic activity during cell proliferation [7], promoting root elongation and lateral root formation in *Medicago truncatula* [15], fruit ripening and quality in tomatoes [16], and drought adaptation in *A. thaliana* [17].

*Brassica napus* has emerged as a valuable model for studying plant responses to environmental stresses, owing to its robust defense mechanisms and metabolic adaptations that ensure survival under extreme conditions [18]. This species faces diverse biotic threats, such as infections by *Botrytis cinerea*, *Sclerotinia sclerotiorum*, and *Leptosphaeria maculans* [19,20,21], as well as abiotic challenges including extreme temperature, drought, and salinity [18,22]. In response to water deficit, abscisic acid (ABA) plays a central role in regulating stomatal closure via guard cell signaling, which involves both reactive oxygen species (ROS) generation and the activation of antioxidant defenses [23]. Transcriptomic studies further indicate that described that external ABA treatments alter the expression of stress- and defense-associated genes, highlighting its function in coordinating antioxidant responses and stress adaptation [24]. Elucidating these tolerance mechanisms is therefore essential for crop genetic improvement.

To date, the *PSK* gene family has been characterized in several plant species, with eight members identified in *Solanum lycopersicum* [9], seven in *Arabidopsis thaliana* [5], sixteen in *Glycine max* [25], fourteen in the *Camellia sinensis* [26], and five in *Lotus japonicus* [27]. In this study, we hypothesized that members of the *BnPSK* gene family are transcriptionally linked to ABA-mediated signaling and abiotic stress responses in *B. napus*. To test this, we performed genome-wide identification of *BnPSK* genes and conducted comprehensive analyses, including phylogenetic relationships, synteny, gene structure, *cis*-acting regulatory elements, and potential post-translational modifications. Expression patterns under hormone and stress treatments were examined using RNA-seq and validated by RT-qPCR. Physiological assays related to antioxidant activity and ROS accumulation were also carried out. Our findings suggest that the peptide hormone BnPSK acts downstream of ABA signaling to fine-tune ROS homeostasis, enhance antioxidant enzyme activity, and mitigate oxidative damage. Together, ABA and *BnPSK* coordinate ROS production and scavenging, thereby optimizing stress adaptation in *B. napus*. This study provides insights into the evolution and potential functions of the *BnPSK* gene family in stress responses.

## 2. Results

### 2.1. Identification, Characterization and Chromosomal Localization of BnPSKs

To identify PSK proteins in *B. napus*, six AtPSK protein sequences were retrieved from TAIR and used as queries to search the *B. napus* genome (Brana ZS V 2.0 pep). After filtering out redundant and incomplete entries, 19 BnPSK proteins containing the PSK superfamily domain (PF06404) were identified subsequently. These proteins ranged from 77 (BnPSK5c and BnPSK5d) to 99 amino acids (BnPSK3c and BnPSK3d) in length (with an average of 90.5 aa), with molecular weights between 8619.71 (BnPSK5c) and 11,591.13 (BnPSK3d) Da. The predicted isoelectric points (pI) varied from 4.46 (BnPSK6a) to 7.79 (BnPSK1c), showing their basic or near-neutral proteins’ nature, and GRAVY scores ranged from −0.737 (BnPSK3c) to 0.009 (BnPSK5a). Moreover, subcellular localization predicted by the online program Plant-PLoc indicated that all BnPSKs are likely chloroplast-localized (Table 1). Lastly, eleven out of nineteen BnPSKs were present on the plus strand (+) while remaining on the minus strand (−) (Table 1). These physicochemical properties, including pI, GRAVY, and molecular weight, may influence the stability, solubility, and subcellular localization of BnPSKs, potentially affecting their accessibility and activity in ABA- and stress-related signaling pathways.

All *BnPSK* genes from *B. napus*, cultivar ZS11, were quantified and the chromosomal distribution of *BnPSKs* was examined on both of the A and C sub-genomes. *BnPSK* genes were distributed in A01 (*BnPSK3a* and *BnPSK4d*), A02 (*BnPSK5c*), A04 (*BnPSK2b*), A06 (*BnPSK1b* and *BnPSK5a*), A08 (*BnPSK6a*), A09 (*BnPSK1c* and *BnPSK4a*), A10 (*BnPSK3d*), C01 (*BnPSK3b* and *BnPSK4c*), C02 (*BnPSK5d*), C03 (*BnPSK5b*), and C04 (*BnPSK2a*), C05 (*BnPSK1a*), C08 (*BnPSK1d* and *BnPSK4b*), and C09 (*BnPSK3c*). Collectively, ten *BnPSKs* were predicted to be localized in A sub-genome and nine *BnPSKs* were found to be localized in C sub-genome of *B. napus* (Figure S1). The nearly balanced distribution between the A and C sub-genomes likely reflects genome duplication events in *B. napus*.

### 2.2. Phylogenetic Relationship and Sequence Similarity/Synteny Among PSKs

**Figure 1:**
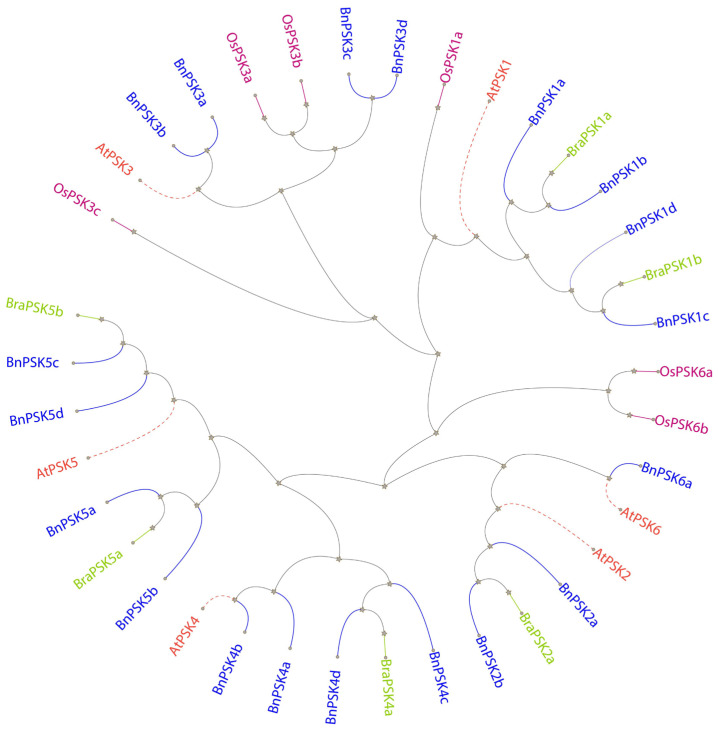
The phylogenetic tree for AtPSK/BraPSK/OsPSk/BnPSK proteins. The Maximum Likelihood (ML) phylogenetic tree of the PSK proteins of *A. thaliana*, *B. rapa*, *O. sativa*, and *B. napus*. The phylogenetic tree was constructed by the MEGA X software with 1000 bootstrap replicates. Distinct colored labels represent PSKs belonging to distinct crops (*A. thaliana*—orange (solid-dashed lines); *B. rapa*—green (solid lines); *O. sativa*—purple (solid lines); *B. napus*—blue (solid lines). Star represents the internal node symbols.

To elucidate the evolution of the BnPSK family, the maximum likelihood phylogenetic tree was generated for six AtPSKs, BraPSK, OsPSK each and nineteen BnPSK proteins (Figure 1). Based on the phylogenetic tree, the BnPSK, BraPSK and OsPSK proteins were named (BnPSK1-19; BraPSK1-6; OsPSK1-6) as respective to their AtPSK homologs. The numbers and distribution of these *BnPSKs* were equal to those of *AtPSKs* (Figure 1), against 6 *AtPSKs*, there were 17 *BnPSKs* identified. Interestingly, all of the respective PSKs were found only in PSKα clade, contained “YIYTQ motif”, meaning none of the respective PSKs were present in the PSKγ clade, PSKε clade, and PSKδ clade. The results showed that AtPSk1 contained four BnPSK, two BraPSk, and one OsPSK orthologs, AtPSK2 contained two BnPSK and one BraPSK orthologs, AtPSK3 contained four BnPSK and three OsPSK orthologs, AtPSK4 contained four BnPSK and one BraPSK orthologs, AtPSK5 contained four BnPSK and two BraPSK orthologs, and AtPSK6 contained one BnPSK and two OsPSK orthologs. This result demonstrated the significant expansion of BnPSKs, BraPSKs and OsPSKs in comparison with AtPSKs.

**Figure 2:**
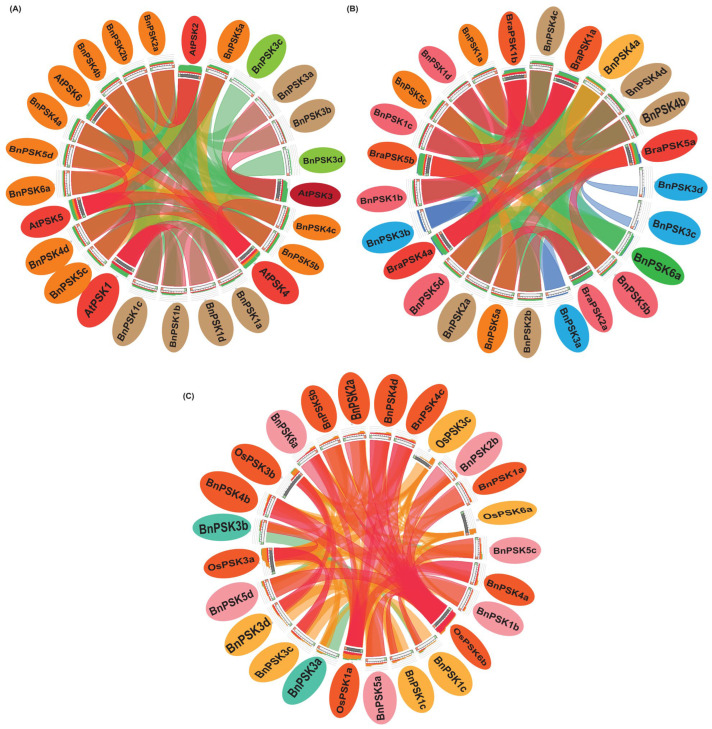
Synteny analysis of *PSKs.* (**A**) *A. thaliana* and *B. napus.* (**B**) *B. napus* and *B. rapa*. (**C**) *B. napus* and *O. sativa*. Ribbon colors indicate sequence identity: blue and light pink (>50%), light green/brown (>75%), orange and red (>99.9999%), illustrating the conservation and divergence of *PSK* gene pairs across four plant species.

To find further conservation among *A. thaliana*, *B. rapa*, *O. sativa*, and *B. napus* PSK proteins, synteny analysis was performed by using the online webserver, Circoletto, that showed strong syntenic conservation, predominantly highlighted by red or orange ribbons (>99%), by light green/brown ribbons (>75%) and by blue and light pink ribbons (>50%), reflecting high sequence identity. This pattern aligns with the evolutionary history of *A. thaliana* as an ancestor of Brassicaceae crops and *B. napus* as a hybrid of *B. rapa* and *B. oleracea*, indicating that most *BnPSKs* have closely related counterparts in *A. thaliana*, *B. rapa*, and *O. sativa*, respectively. Ultimately, the high level of similarity suggests limited divergence and potential functional conservation among these homologs (Figure 2A–C).

### 2.3. Analysis of Structural Features of BnPSKs

**Figure 3:**
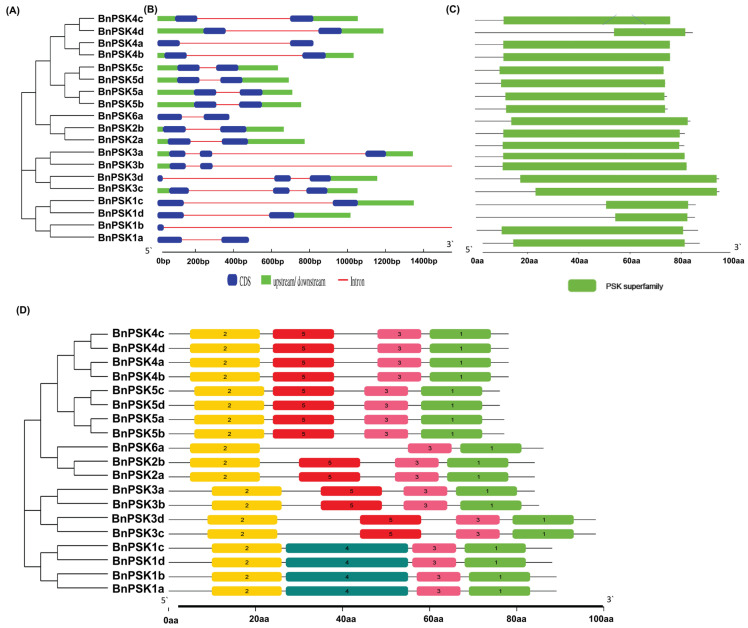
Phylogeny, exon–intron structures of 19 *B. napus PSKs*, conserved domains and motifs. (**A**) Phylogenetic tree constructed among BnPSK proteins. (**B**) The constituents of *BnPSK* genes are indicated in differentially colored shapes. CDS: blue box; upstream/downstream: green box; introns: red lines. (**C**) Conserved domain analysis in the genomes of *B. napus* cultivar ZS11 through SMART database. (**D**) Motif profile among BnPSKs. Each motif is represented in different colors using TBtools (v2.084). Motif 1: active PSK peptide core [28]; Motif 2: functions as signal peptide [6]; Motif 3: functions infolding and proteolytic release of the mature peptide [14]; Motif 4: regulatory propeptide region that controls proteolytic processing [29]; Motif 5: regulates PSK precursor processing and maturation [6].

The phylogenetic tree was constructed among all BnPSK proteins to show evolutionary relationship among them (Figure 3A). Gene structures along with the protein domains were also analyzed for all the *PSK* genes (Figure 3B,C). All *PSK* genes contained only one intron as well as two CDS regions, with four exceptions, *BnPSK3a*, *BnPSK3b*, *BnPSK3c*, and *BnPSK3d*, having two introns and three CDS regions.

Next, we investigated the gene sequence and structure of *BnPSKs* to gain a clearer understanding of conserved structure, physiological function, and evolutionary divergence. A conserved motif analysis revealed that 18 BnPSKs contained four motifs, while BnPSK6a had three motifs (1, 2 and 3), and most of them had a similar order (Figure 3D). Four PSKs (BnPSK1a, BnPSK1b, BnPSK1c, and BnPSK1d) that confined all of the motifs except ‘motif 5’ that was absent in remaining all of the BnPSKs. The most important motif that is the key motif for PSKs characterization (YIYTQ; motif 1) was found predicted in each and every single BnPSK protein (Figure S2) which is responsible for functions in other crops’ PSKs [25,26].

To further investigate the structure of the *BnPSK* genes, a multiple sequence alignment was performed. As shown in Figure S1, the *B. napus* PSK proteins contain the conserved functional domain including the pentapeptide motif (YIYTQ), involved in plant defense mechanisms [28] and highly conserved “cystein (C) and glutamate (E)”.

The motif logos and their consensus also showed the conservativeness among them and motif ‘4’ was present only in AtPSK1-*B. napus* orthologs (BnPSK1-BnPSK4). These specific motif logos were generated by using weblogo3 (v3.7). The bit scores for each position in the specific motif logos were generated on the left side of the column (Figure S3). Overall, these results illustrated the presence of important motifs indicating the importance of BnPSKs in plant growth functions, biotic/abiotic stress resilience and regulation through phytohormones under stress conditions.

### 2.4. Investigation of Cis-Acting Regulatory Elements (CREs) in the Promoter of BnPSKs

**Figure 4:**
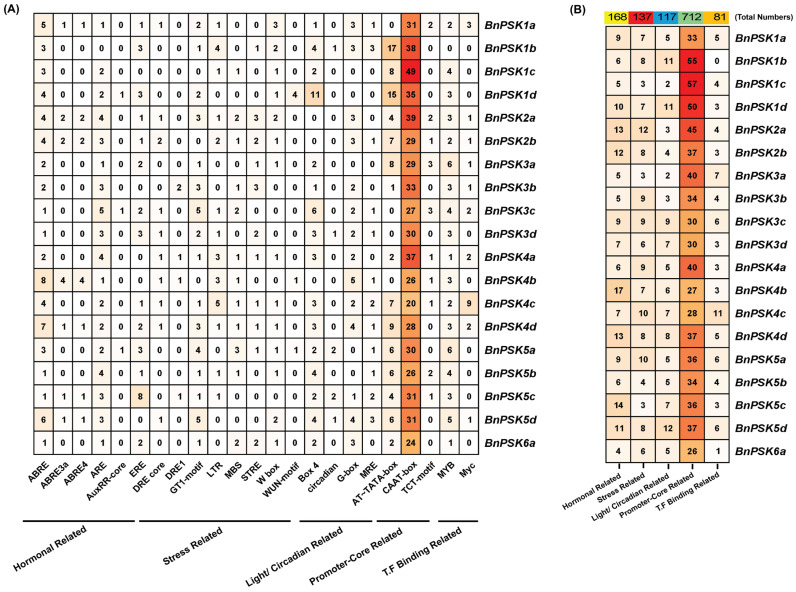
Predicted *cis*-acting regulatory elements (CREs) in *PSK’s* promoters. (**A**) Major hormone-related, stress-related, light-/circadian-related,-promoter-core-related and T.F-binding-related CREs predicted in *BnPSKs*’ promoters were shown. Individual CREs were provided. (**B**) CREs were categorized extensively. The bubble heat map showing the number of total elements was prepared using TBtools (v2.084).

To elucidate the preliminary functions of *BnPSKs*, *cis*-acting regulatory elements were predicted in the upstream region, around 2 kb. The predicted CREs have been categorized into several aspects including hormonal-related (6), stress-related (8), light-/circadian-related (4), promoter-core-related (3) and transcriptional-factor-binding-related (2) (Figure 4A,B). The presence of most of the stress- or hormonal-related *CREs* in their promoter signified the adaptation of *BnPSKs* in plant stress management. Additionally, the presence of core-promoter elements and T.F binding CREs also signified their importance in regulating gene functions. ABRE was found abundantly in *BnPSK4b* (62) in all of the *BnPSKs*, and one *BnPSK4b* contained the maximum eight ABRE CRE. From stress-related CREs, two *PSKs* including *BnPSK3c* and *BnPSK5d* contained maximum (five) ‘GT1-motif’ from the overall 62-GT1-motif present in all *BnPSKs*, and DRE1 was found to be the minimum (only five) in all of the *BnPSKs*. One of the light-/circadian-related elements, BOX 4, was found in almost all of the *BnPSK* abundantly (53). *BnPSK1d* was found enriched with BOX 4 *cis*-acting regulatory element (11). Some of the frequently found CREs including CAAT-Box (593) and AT~TATA-box (102) were found richly in most of these genes. It is noteworthy that the presence of these CREs showed their importance in regulating gene functions. Some other CREs, including transcriptional factor binding sites (Myb- Myc), indicated their transcriptional regulation via their promoter activity (Figure 4A,B). Collectively, the presence of ABA and stress-related CREs in the promoters of BnPSKs emphasized their contribution to ABA-mediated drought stress tolerance in *B. napus* plants.

### 2.5. PTM Sites Prediction in BnPSK Proteins

**Figure 5:**
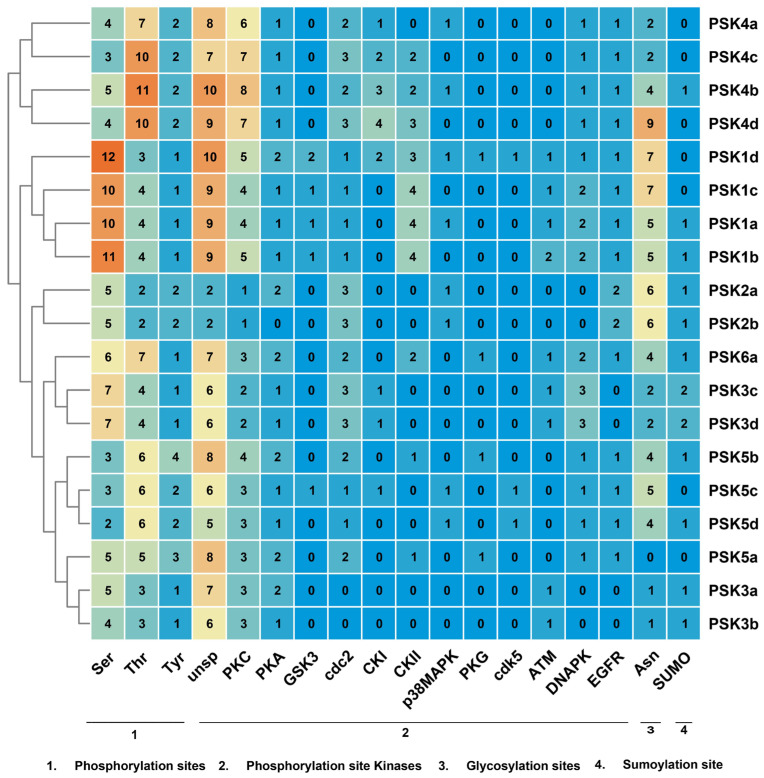
Predicted phosphorylation, glycosylation, and sumoylation sites of BnPSK proteins. Bubble colors (blue to red) indicate the number of PTM sites. unsp, unspecified phosphorylation; PKC, protein kinase C; PKA, protein kinase A; GSK3, glycogen synthase kinase 3; CDC2, cell division cycle protein 2; CKI, casein kinase 1; CKII, casein kinase 2; P38MAPK, P38 mitogen-activated protein kinase; PKG, protein kinase G; CDK5, cyclin-dependent kinase 5; DNAPK, DNA-dependent protein kinase; EGFR, Epidermal Growth Factor Receptor. The bubble heat map showing the number of PTM sites was prepared using TBtools (v2.084).

The phosphorylation of serine, threonine, and tyrosine residues in BnPSK proteins was predicted. In general, all predicted proteins contained phosphorylation sites for each of these three amino acids. BnPSK1d had the highest number of potential serine phosphorylation sites (12), BnPSK4b and BnPSK4c had the most threonine sites (11 and 10, respectively), and BnPSK5a and BnPSK5b had the maximum tyrosine sites (3 and 4, respectively) (Figure 5). These conclusions were based on the prediction which suggested that BnPSK proteins are likely to be regulated by phosphorylation as no. of phosphorylation sites, and their kinases (PKC and PKA) were predicted abundantly in BnPSKs. For example, BnPSK4b and BnPSK4c contained the maximum eight and seven protein kinase C (PKC), respectively, while several BnPSKs including BnPSK1d, -2a, -3a, -5a, -5b, and -6a, contained the maximum two of the protein kinase A (PKA) in their proteins. Overall, the phosphorylation levels of UNSP (134) and protein kinase C (74) were the highest in BnPSK proteins, while those of PKG (4) and cdk5 (3) were extremely low. Moreover, on the basis of linkage between amino acid and sugar molecule, glycosylation site can be classified into four main types including N-linked glycans, GPI anchors, C-mannosylation and O-linked glycans. Analysis of glycosylation patterns in PSK proteins identified O-linked glycans, which is specific for the binding of the sugar molecule with the hydroxyl group of serine or threonine. Among the PSK proteins, BnPSK4d contained the highest number of nine “Asn” residues, while BnPSK5a had none of the “Asn” residue (Figure 5). For sumo-sites prediction analysis, BnPSK3c and BnPSK3d had the maximum two predicted sumo-sites while seven out of nineteen BnPSKs (BnPSK1c, -1d, -4a, -4c, -4d, -5a, and -5c) did not contain even a single sumo-site. These results indicated that all of the BnPSKs were predicted to be glycosylated and some of them were sumoylated, which could be more effective in stress-related phenomena in plants.

### 2.6. PPI Analysis

**Figure 6:**
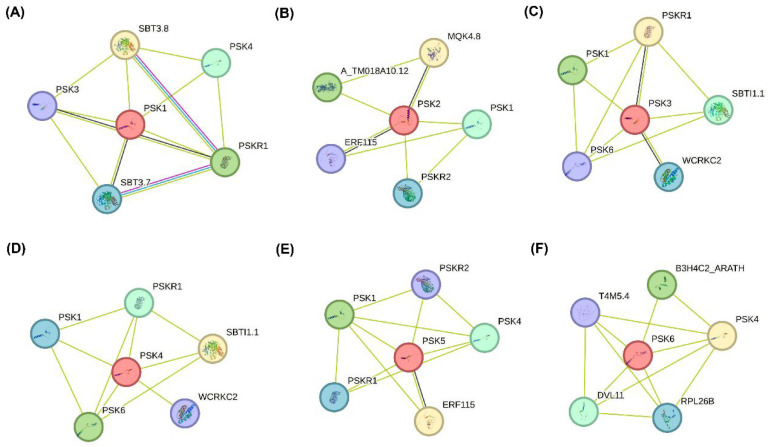
Prediction of protein–protein interactions (PPIs) between *B. napus* PSKs and other stress-related proteins by using the STRING database. (**A**) PPI network among AtPSK1 (homolog of BnPSK1a/b/c/d). (**B**) PPI network among PSK2 (homolog of BnPSK2a/b). (**C**) AtPSK3 (homolog of BnPSK3a/b/c/d). (**D**) AtPSK4 (homolog of BnPSK4a/b/c/d). (**E**) AtPSK5 (homolog of BnPSK5a/b/c/d). (**F**) AtPSK6 (homolog of BnPSK6a) and other stress-responsive proteins.

A protein–protein interaction (PPI) network was constructed using the STRING database to investigate the key involvement of BnPSK proteins through their interactions with other respective proteins based on both known experimental data and predicted interactions. To predict the PPI network, homologous proteins from *A. thaliana* were used as it is considered as a model plant and all of its interacting partner proteins are also well-reported. Secondly, it might be possible that the orthologs of AtPSKs from *B. napus* possibly had quite similar functions in response to stresses. As shown in Figure 6A–F, the homologs AtPSK1, AtPSK2, AtPSK3, AtPSK4, AtPSK5, and AtPSK6 (corresponding from BnPSK1a to BnPSK6a) shared several potential interacting proteins, including subtilisin-like serine proteases (SBT3.8), involved in osmotic stress tolerance in *A. thaliana* [17], and PSK3 and PSK4, involved in biotic stress tolerance [30]. The interaction network highlights PSK peptides (PSK1–PSK6) and their receptors (PSKR1, PSKR2) as central components potentially involved in ABA-mediated drought tolerance in *B. napus*. Transcription factors such as ERF115 and WRKY-related proteins (e.g., WCRKC2) are connected to this network and are known regulators of abiotic stress responses and ABA signaling [31,32]. Additionally, the kinase-like protein MQK4.8 may contribute to signal transduction, consistent with the role of kinase cascades in integrating hormonal and stress signals [33]. These findings support a coordinated role of peptide signaling, transcriptional regulation, and kinase activity in modulating drought stress adaptation, while some of the experimental verifications wait for further studies.

### 2.7. Expression Profiling of BnPSKs

#### 2.7.1. Expression Dynamics in Different Plant Parts Using RNA-sequence Data

**Figure 7:**
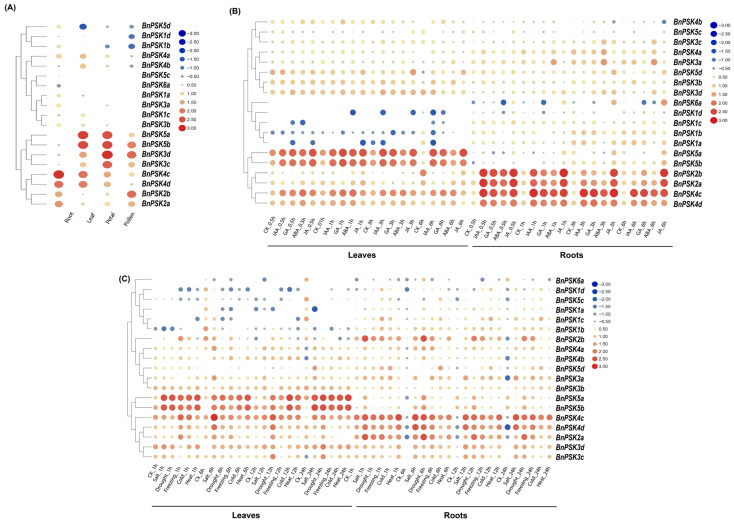
Quantification of transcriptional regulation of *BnPSKs* using RNA-seq data. (**A**) Expression patterns of *BnPSK* genes in four different tissues of *B. napus* plants. Expression responses of *B. napus* plants after (**B**) IAA, GA, ABA, JA applications in plant roots and leaves. (**C**) Salt stress, drought stress, freezing stress, cold stress, heat stress in plant roots and leaves. The size and color of the circles represent high and low expression levels, with red indicating high expression or upregulation and dark blue indicating low expression or downregulation. The TPM values were converted into log2FC before plotting the heatmap.

The expression analysis of *BnPSKs* was investigated using publicly available RNA-sequence data from the BnIR (*Brassica napus* multi-omics information resource). An analysis of the *BnPSK* expression values (Transcripts Per Kilobase Per Million mapped reads, TPM value converted into log2 values) revealed different expression patterns in four tissues (root, leaf, petal, pollen). The results showed that some of the *BnPSKs* are expressed in the respective plant tissues, while the remaining ones did not express well. Although, the expression of some genes was tissue-specific. For example, *BnPSK4c* expressed in roots, *BnPSK5a* and *BnPSK5b* comprised the maximum expression in plant leaves, *BnPSK3c* and *BnPSk3d* expressed relatively higher in plant petals. *BnPSK* genes of different types (a, -b, -c, -d) and from different sub-genomes (A/C) in *B. napus* often exhibit tissue-specific expression patterns, reflecting sub-genome expression bias and functional specialization that allow for a fine-tuned regulation of growth and development across various tissues (Figure 7A).

#### 2.7.2. Expression Profiling Using RNA-Sequence Data Following Phytohormone Applications

For a better understanding of the regulatory role of *BnPSKs* in plant hormone signaling pathways, the expression of *BnPSKs* was analyzed by RNA-sequence data after different hormone application (IAA, GA, ABA, and JA). We analyzed the expression of *BnPSKs* by using RNA-sequence data in plant roots after hormone application. The *BnPSK4c* and *BnPSK2b* exhibited maximum expression just after 3 h and 1 h of JA application (log2FC value ~2.75 and ~2.76), respectively. In plant leaves, the expression of *BnPSK5a* exhibited a strong trend after 1 h of GA application (log2 value ~2.47) and after 6 h of JA application (log2 value ~2.41) (Figure 7B).

#### 2.7.3. Transcript Profiling Using RNA-Sequence Data Under Abiotic Stresses

To further elucidate the stress response mechanism of *BnPSKs*, the expression of *BnPSKs* was analyzed by using RNA-sequence data under different abiotic stress conditions by converting the expression value into log2FC value. We analyzed the expression of *BnPSKs* by using RNA-sequence data in plant roots. The *BnPSK4c* had maximum expression just after 1 h of heat stress (~1.5-Fold), 6 h after salt stress (more than ~1-Fold) and after 6 h of drought treatment (~1.5-Fold). In plant leaves, the expression of *BnPSK5a* exhibited a strong trend after 24 h of heat stress treatment (~around ~2-fold), 24 h after drought stress (~1.5-fold) and after 12 h of cold treatment (~1-fold). The *BnPSK5b* showed the highest maximum expression in plant leaves after 24 h of salt stress treatment (approximately ~1.5-fold), 24 h after drought stress (more than ~1-fold), and after 12 h of cold treatment (around ~2-fold). The heat map revealed an increased expression of *BnPSK4c*, *BnPSK5a*, and *BnPSK5b* in response to heat, drought, cold and salt stress in plant roots and leaves, respectively (Figure 7C). Data were statistically analyzed by Student’s *t*-test and they were found at par statistically (*p* < 0.05).

#### 2.7.4. Transcriptional Regulation of BnPSKs in Plant Tissues and Following ABA Application Under Drought Stress via RT-qPCR

**Figure 8:**
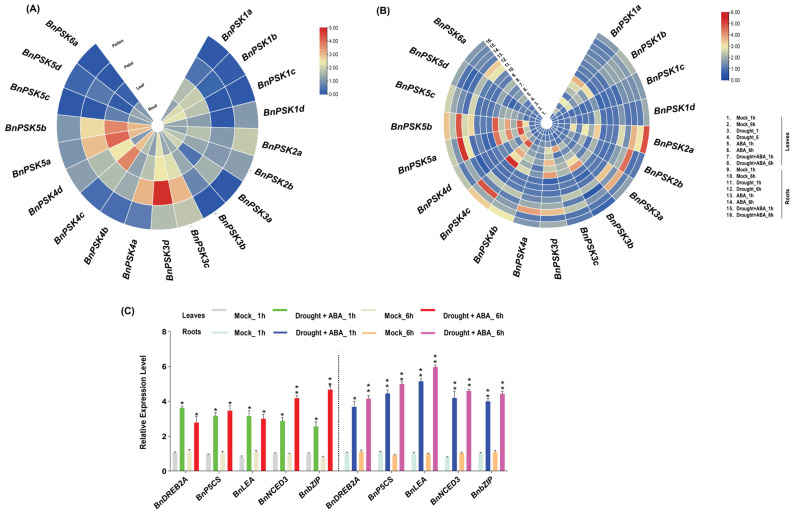
Tissue-specific expression profiles of *BnPSK* genes and their responses to ABA and abiotic stresses. (**A**) Expression patterns of *BnPSKs* in four tissues. All values were expressed relative to the expression levels of reference genes using formula 2^−ΔΔCt^ (*n* = 3). (**B**) Expression patterns of the *BnPSKs* at 1 and 6 h after being treated with drought stress and ABA application. All treatments mentioned on the right side of the figure. Different colors (blue to red) indicate significant (*p* < 0.05) up/down-regulated expression, generated using TBtools (v2.084). (**C**) Relative expression of selected ABA-responsive, drought-related genes, normalized to *BnActin* using the 2^−ΔΔCt^ method (n = 3). Asterisks denote statistical significance (* *p* ≤ 0.05, ** *p* ≤ 0.01; Student’s *t*-test). All experiments were repeated three times with similar results.

The ABA- and stress-induced expression of BnPSK4c, -5a and -5b, may be related to the presence of ABRE CREs in their promoters and predicted PTMs, suggesting a potential mechanism for transcriptional and post-translational regulation under stress conditions. To confirm the both, accuracy of RNA-seq data and the presence of ABA-responsive *cis*-acting regulatory elements, the relative expression was quantified in plant tissues via RT-qPCR using log2FC value. The quantification of these genes showed a significant transcription level in plant roots (*BnPSK4c*) and plant leaves (*BnPSK5a* and *BnPSK5b*), about ~2-fold, ~3-fold, and ~2.5-fold, respectively (Figure 8A). Surprisingly, one gene, *BnPSK3d*, expressed significantly in petals of *B. napus* plants. Subsequently, to substantiate the obtained results for ABA-regulated transcriptional activity, RT-qPCR analysis showed that *BnPSK4c*, *BnPSK5a*, and *BnPSK5b* expressed significantly in plant leaves and roots after ABA application at 6h post treatments, about ~4-fold and ~5-fold, respectively, and under drought + ABA combined treatment about ~5-fold. The obtained results indicated their involvement in plant hormone signaling pathways in mitigating drought stress in rapeseed plants (Figure 8B).

To validate the effects of ABA in drought-tolerance in *B. napus*, some of the tolerance-inducing genes (*BnDREB2A*, *BnP5C5*, *BnLEA*, *BnNCED3*, *BnbZIP*) have been examined via RT-qPCR. The obtained results demonstrated that the expression of tolerance inducing genes after combined effect of ABA + drought was significantly high as compared to other treatments (Drought and ABA, separately). The analysis declared that respective genes exhibited significant upregulation under ABA-induced drought stress in *B. napus’* leaves, with *BnbZIP* showing an approximately ~4-fold increase at 6 h post-treatment (hpt), *BnNCED3* and *BnP5CS* peaking at 3~4-fold and 2~3-fold, respectively, at 6 hpt. The expression of former genes was relatively higher in plant roots than leaves. For example, *BnLEA* reached ~5-fold at 6 hpt, and *BnP5CS* displayed a ~4-fold increase at 6 hpt (Figure 8C), while one gene (*BnDREB2A*) expressed relatively lower than the remaining all genes. The results depicted that ABA could induce transcriptional activity in rapeseed plants (leaves and roots) to minimize the effects of drought stress.

### 2.8. Impact of Drought Stress on B. napus Crop: Evaluation of Induced Resistance

**Figure 9:**
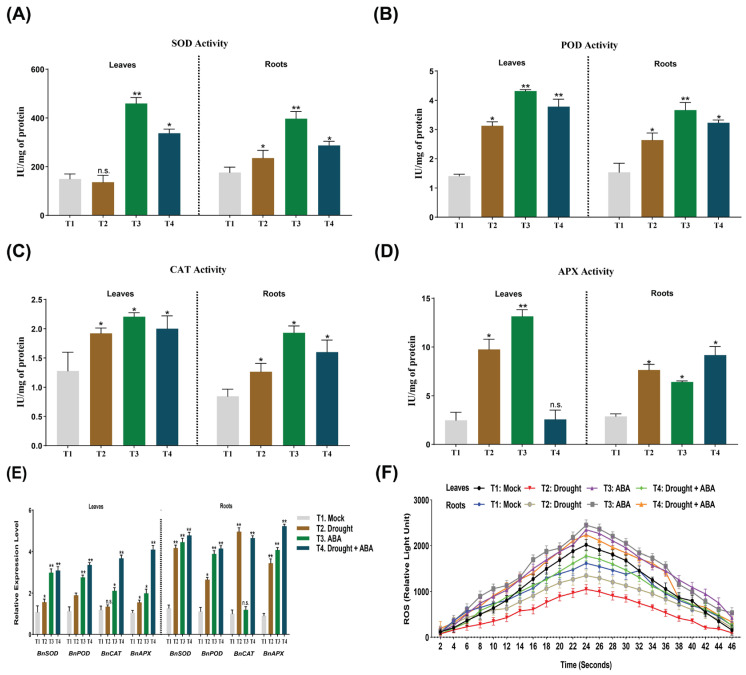
Effect of ABA on antioxidant enzymes in one-month-old *B. napus* plants under drought stress. (**A**) Effects of abscisic acid (ABA) on superoxide dismutase (SOD) by using drought-stress treated plants’ leaves and roots. (**B**) Peroxidase (POD). (**C**) Catalase (CAT). (**D**) Ascorbate peroxidase (APX). (**E**) Transcriptional activation of *BnSOD*/*BnPOD*/*BnCAT*/*BnAPX* genes in response to abscisic acid (ABA) application under drought stress. Gene expression of four *BnSOD*/*BnPOD*/*BnCAT*/*BnAPX* genes by qRT-PCR, normalized to *BnActin* using the 2^−ΔΔCt^ method (n = 3). Asterisks denote statistical significance (* *p* ≤ 0.05, ** *p* ≤ 0.01, n.s., not significant; Student’s *t*-test). (**F**) Reactive oxygen species (ROS) measured in oilseed rape leaf disk assay after the addition of 150 µM ABA (under drought stress) or water (Mock). The dynamics of ROS production as total photon counts over 45 min have been shown (as the mean ± SE). RLU, relative light units. All experiments were repeated three times with similar results.

ABA, as shown in previous studies, plays a magnificent role in mitigating abiotic stress, especially drought stress. In current study, phytohormone, ABA, was also considered as a potential candidate to enhance nutrient accumulation in plant roots and leaves. Compared to untreated control plants, ABA treatment significantly augmented the antioxidant activity (SOD, POD, CAT, and APX) in roots and leaves of oilseed plants (Figure 9A–D). In comparison, drought-stress-treated plants exhibited a disruption in ionic homeostasis, with substantial induction in antioxidant enzymatic activity. In case of application of ABA, maximum SOD, POD, and CAT activity were recorded in plant leaves (459, 4.31 and 2.2 IU/mg, respectively) while in plant roots, they were recorded as 296, 3.66, and 1.93 IU/mg, respectively (2~5 fold). In contrast, maximum APX activity was recorded to be 9.16 IU/mg in plant roots after ABA application under drought stress. Moreover, the antioxidant activity reduced drastically after drought stress both in plant leaves (SOD: 136; POD: 3.15; CAT: 1.91; APX: 9.75 IU/mg) and roots (SOD: 235; POD: 2.6; CAT: 1.26; APX: 7.64 IU/mg). ABA also enhanced the antioxidant enzymatic activity under drought stress both in plant leaves (SOD: 337; POD: 3.78; CAT: 2.0; APX: 2.56 IU/mg) and roots (SOD: 287; POD: 3.22; CAT: 1.60; APX: 9.16 IU/mg). Therefore, it might be concluded that the application of ABA mitigated the drought stress in *B. napus* plants to some extent.

We also examined the *BnSOD*, *BnPOD*, *BnCAT*, and *BnAPX* expression levels after ABA application with *B. napus* plants’ leaves and roots under drought stress conditions. All of the above-mentioned genes were expressed significantly after drought stress and ABA application. *BnSOD*, *BnPOD*, *BnCAT*, and *BnAPX* were induced significantly after the combined (drought and ABA) application (Figure 9E). The relative expression level of all genes was significantly higher in plant roots (4~5-fold) as compared to plant leaves (3~4-fold). These results demonstrated that all genes involved in antioxidant activity have significant expression showing that ABA could accelerate tolerance in rapeseed plants under drought stress conditions.

ROS signaling was also measured (as RLU) in rapeseed plants to see the role of ABA in mitigating the drought stress by minimizing ROS activity (Figure 9F) by recording the ROS activity for more than 40 s. Maximum ROS activity was recorded in plant roots (2449 RLU) and leaves (2338 RLU) after ABA application and then declined after 24 s to 534 RLU and 427 RLU, respectively, just after 46 s. On the contrary, ROS activity reached its maximum in rapeseed plant leaves (1049 RLU) and roots (1338 RLU) only under drought stress conditions, which is significantly reduced as compared to ABA-treated *B. napus* plants. Moreover, ROS signaling was recorded in between both former treatments. ROS activity improved in drought-stress-treated plant’s leaves (1779 RLU) and roots (2239 RLU) as compared to only stress-treated plants.

## 3. Discussion

The *BnPSK* gene family encodes a class of sulfated peptides predominantly present in plants and is involved in regulating diverse developmental pathways and stress responses. The *PSK* genes have been systematically studied in many plants [6], but not in *B. napus* plants. Therefore, we conducted a comprehensive analysis of the *BnPSK* gene family in the rapeseed plant to determine the characteristics of *BnPSK* genes. It is reported that genes within the same clade of the phylogenetic tree may encode sequences with similar biological functions [34]. Here, we surveyed the *B. napus* plant genome using various bioinformatics methods and obtained 19 members of the *BnPSK* gene family. A set of evidence supports them to be potentially functional *PSKs* [35]. The 10 *BnPSK* genes were distributed in sub-genome “A” and 9 *BnPSKs* were found in abundance in sub-genome “C” (Table 1), which may suggest an evolutionary adaptation in rapeseed plants to enhance their resilience to environmental changes. An interspecific synteny analysis among *B. napus*, *A. thaliana*, *O. sativa*, and *B. rapa* further elucidated the homologous relationship within the *PSK* gene families (Figure 2). The variation in exon–intron structures is closely linked to gene duplication and functional diversity. Genes that lack introns can rapidly respond to changes in environmental factors and are primarily induced by stress [34]. Only four of the *BnPSK* genes (*BnPSK3a*/*b*/*c*/*d*) contained two introns each while the remaining ones all have only one intron (Figure 3B). They all consist of a PSK-domain and most of them had transmembrane regions except BnPSK2a, BnPSK2b, BnPSK3c, and BnPSK6a (Table 1; Figure 3C). Furthermore, all identified BnPSKs belong to the PSK-α group and contain the functional pentapeptide conserved motif (YIYTQ) (Figure 3D) as in previous reports [27,35]. Only four PSKs, BnPSK1a/b/c/d, exhibited motif ‘4’ that was not predicted in other BnPSKs. All BnPSKs contained the conserved functional domain of the PSK family.

In addition, the BnPSK proteins were predicted to be localized to chloroplast as some of the previously reported CsPSKs [26] (Table 1). All of the predicted BnPSKs were considered as acidic or near-neutral proteins on the basis of their PI (iso-electric point). Additionally, the number of *BnPSK* genes is significantly higher as compared to other species, such as the model plant species tomato [36] and *Arabidopsis* [5]. Interestingly, promoter-core-related CREs (specifically, CAAT-Box) were found abundantly in *BnPSK1c* (49) while predicted to be at a minimum in *BnPSK4c* (20). In total, stress-related CREs were also found in abundance (137 in all BnPSKs) and among them, *BnPSK2a* exhibited the maximum 12 of stress-related CREs (Figure 4). These findings elaborated the response of *BnPSKs* in biotic/abiotic stresses and plant hormone-related stress tolerance. Notably, PTM prediction analysis concluded that all of the BnPSK proteins are likely to be phosphorylated and/or glycosylated, while some of them can also be sumoylated (Figure 5), indicating their role in important plant molecular processes as previously reported PSKs [26]. PPI analysis claimed that PSKs are closely linked with each other and might be involved in regulating plant stress tolerance synergistically (Figure 6) [18].

Our expression analyses results showed significantly expressed *BnPSK* genes in some of the respective plant parts. For example, *BnPSK4c* and *BnPSK4d* are expressed relatively higher (upregulated) in plant roots as compared to other plant parts while *BnPSK5a* and *BnPSK5b* are expressed substantially (upregulated) in plant leaves and petals. Lastly, *BnPSK2b* and *BnPSK3d* are upregulated in plant pollens that showed a diversified expression pattern of *BnPSKs* (Figure 7A and Figure 8A) among all the plant parts. This concludes the tissue-specific expression of *BnPSKs* that seems to be aligned with previous studies [25,34] in which *CsPSKs* and *TaPSKs* have tissue-specific gene expressions. According to RNA sequence data analyses, *BnPSK4c* and *BnPSK5a*/*BnPSK5b* displayed distinct expression patterns; a similar trend was observed between them related to abiotic stress and phytohormone application (Figure 7B and Figure 8B,C).

In addition, gene expression analysis provides valuable insights into gene distribution and function, helping us to better understand their potential roles in various biological processes [37]. In *Arabidopsis*, *PSK1* exhibited expression across all cell layers, with stronger expression in the epidermis, while *PSK2*, *PSK3*, *PSK4*, and *PSK5* were primarily detected in the central cylinder, indicating their potential key roles in root growth and development [38]. Remarkably, in the present study, the transcriptional level of three *BnPSK* genes (*BnPSK4c*, *BnPSK5a*, and *BnPSK5b*) was significantly induced following drought stress treatment and ABA application, indicating their potential role in plant growth and development as previously claimed for *TaPSKs* and *GmPSKs* [30,34]. These results suggested that the *BnPSK* gene family in *B. napus* may have undergone functional divergence during evolution, likely in response to selective pressures from the external environment.

Transcriptomic and RT-qPCR analyses revealed that several *BnPSK* genes, particularly *BnPSK4c*, *BnPSK5a*, and *BnPSK5b*, exhibited altered expression patterns in response to drought stress and exogenous ABA treatment. These expression changes indicate a potential association between *BnPSKs* and ABA-responsive stress signaling pathways. However, the observed transcriptional responses alone do not establish direct functional roles for *BnPSKs* in ABA-mediated drought tolerance. In parallel, ABA application was associated with enhanced antioxidant enzyme activities (SOD, POD, CAT, and APX) (Figure 9A–D) and reduced ROS accumulation in both leaves and roots, consistent with the well-documented role of ABA in drought stress adaptation (Figure 9F) [23]. While these physiological responses occurred alongside changes in BnPSK expression, the data primarily support a correlative relationship. One latest study revealed that the exogenous applications of ABA and its signaling are associated with the regulation of stress and defense genes, including transcriptomics, as well as the endogenous role of ABA in antioxidant and stress response mechanisms also being noticed [24]. Taken together, these findings suggest that BnPSKs may be involved in ABA-associated stress responses, but further functional studies are necessary to elucidate their specific regulatory roles and molecular mechanisms in drought tolerance.

## 4. Materials and Methods

### 4.1. Identification and Characterization of BnPSK Genes

The PSK protein sequence of *A. thaliana* was downloaded from The *Arabidopsis* Information Resource (TAIR, https://www.arabidopsis.org, accessed on 28 June 2024). The *B. napus* genome and protein sequence were downloaded from the Brassicaceae database (http://www.brassicadb.cn/, accessed on 3 July 2024). Using the AtPSK protein sequence as a reference, a comparative genome-wide search in the Brassicaceae database was conducted using the blast program. Subsequently, all predicted BnPSK proteins were checked and subjected to SMART Database (https://smart.embl-heidelberg.de/, accessed on 15 July 2024) and MEME (https://meme-suite.org/meme/, accessed on 17 July 2024) analysis to confirm conserved motifs and domains. The physicochemical properties of the BnPSK proteins were analyzed using Protparam (https://web.expasy.org/protparam/, accessed on 20 July 2024) [38]. Subcellular localization was predicted using the online program Plant-PLoc (http://www.csbio.sjtu.edu.cn/bioinf/plant/, accessed on 3 August 2024). Furthermore, the chromosome location of BnPSKs was identified using the BRAD database [18].

### 4.2. Phylogenetic Relationship and Synteny Analysis

A phylogenetic tree was constructed in MEGA X (v10.2.2) using the Neighbor-Joining method with 1000 bootstrap replicates and a 95% site coverage cut-off for gap handling to evaluate evolutionary relationships and subgroup classification among *B. napus*, *B. rapa*, *O. sativa*, and *A. thaliana* PSK proteins [39]. Syntenic relationships among *PSK* genes from *B. napus*, *B. rapa*, *O. sativa*, and *A. thaliana* were examined using genome annotation files retrieved from public databases (Circoletto, https://bat.infspire.org/circoletto/#news, accessed on 12 December 2024) used for synteny analysis to identify collinear gene pairs based on sequence homology [40].

### 4.3. Sequence Alignment and Gene Structure Analysis of BnPSK Genes

The amino acid sequences of BnPSK were aligned using ClustalW (version 2.1), and the results were visualized with ESPript 3.0 (https://espript.ibcp.fr/ESPript/cgi-bin/ESPript.cgi, accessed on 6 August 2024). The coding sequences (CDSs) and Untranslated Regions (UTRs) of the *BnPSK* genes were extracted based on the genome annotation information of *B. napus*. TBtools (v2.084) was used to display the phylogenetic trees, motif, and domain patterns. GSDS (https://gsds.gao-lab.org/Gsds_help.php, accessed on 1 January 2025) was used to predict the structures of *BnPSKs*. Gene structures, including exon–intron organization, were analyzed by aligning coding sequences with genomic DNA using the GSDS tool [41].

### 4.4. Prediction of Cis-Acting Regulatory Elements (CREs) and Post-Translational Modification Sites

For promoter profiling, 2 kb upstream sequences from each *BnPSK* gene were retrieved from the BRAD database and analyzed using the plantcare database (https://bioinformatics.psb.ugent.be/webtools/plantcare/html/, accessed 29 January 2024) to identify putative *cis*-acting regulatory elements (CREs) associated with stress-, hormone-, circadian-/light-, core-promoter-related, and transcriptional-factor-binding-related *cis*-acting element [42].

Post-translational modification (PTM) sites in BnPSk proteins were predicted to assess their potential roles in different stress-related responses. N-glycosylation sites were identified using NetOGlyc (http://www.cbs.dtu.dk/services/NetOGlyc/, accessed 29 January 2024), sumoylation sites at lysine residues were predicted using Sumoplot webserver (http://deepsumo.renlab.org/, accessed 1 February 2024), and phosphorylation sites at serine, threonine, and tyrosine residues were predicted using NetPhos; http://www.cbs.dtu.dk/services/NetPhos/, accessed 4 February 2024) [43].

### 4.5. Protein Interaction Network Analysis

The protein–protein interaction (PPI) analysis of Brassicaceae PSK proteins was conducted using the *Arabidopsis* model in the STRING database [18].

### 4.6. Plant Materials and Experimental Conditions (RNA-Seq and RT-qPCR)

The seeds of the commercial cultivar, Zhongshuang11, of *B. napus* were obtained from the Institute of Seed Science, College of Agriculture and Biotechnology, Hangzhou, Zhejiang Province, China. Plants were cultivated in growth chambers at 22–23 °C under 14 h/10 h (light/dark) photoperiod in 250 mL plastic pots containing a potting media (peat moss/perlite = 4:1) for four weeks. The plants were fertilized from a commercial fertilizer, “Kang Pu Jin” 20–20 20ofN-P2O5-K2O Mg TE (COMPO Expert GmbH, Krefeld, Germany) and watered as necessary in the growth chamber. After four weeks, plants were subjected to various treatments, including 200 mM NaCl, drought (22 °C, 25% PEG-6000 for 24 h) [17], freezing (−4 °C for 4 h after 24 h of cold acclimation), cold (4 °C for 24 h) [43], heat (35 °C for 24 h) [44]. Phytohormones were also applied at the same plant age at different concentrations, i.e., 1 μM IAA [44], 100 μM GA3 [45], 50 μM MeJA [46], ABA 150 μM [47]. After respective treatments, samples were taken and put in a −80 °C refrigerator to assess gene expression, nutrients deposition, and antioxidant defense mechanisms later. Three biological replicates were performed.

### 4.7. Analysis of BnPSK Genes Expression Pattern Using RNA Seq Data

RNA sequencing data of *B. napus* plants were downloaded from the *B. napus* multi-omics information resource (BnIR; https://yanglab.hzau.edu.cn/BnIR/expression_zs11, accessed on 12 July 2024). The expression abundance of *BnPSK* genes in different tissues, hormonal application, and abiotic stresses was represented using Transcripts Per Million (TPM) values and submitted to TBtools (v2.084) to generate heatmaps. For hormone treatments (ABA, JA, GA, and IAA), samples at 1 h, 3 h, and 6 h represent early and intermediate signaling responses following hormone perception. For abiotic stresses, sampling at 1 h, 6 h, 12 h, and 24 h allows for the assessment of immediate stress responses, as well as later transcriptional adjustments associated with stress acclimation. All of the heatmaps were normalized by log2FC value. We set the criteria for significantly differentially expressed (DEGs) as that of *p* < 0.05 and fold change >2 or <2.

### 4.8. RNA Extraction and cDNA Synthesis for RT-qPCR

The extraction of total RNA from the leaf samples of *B. napus* plants was performed using an RNA isolation kit (Takara, Shiga, Japan) [48]. Afterwards, the extracted RNA was assessed for quality and quantity using a Nano Drop-1000 spectrophotometer (Thermo Scientific, Waltham, MA, USA). To convert isolated RNA into cDNA, the PrimeScript™ RT reagent kit (Takara, Shiga, Japan) was utilized [49,50]. Then, qRT-PCR was performed using SYBR^®^ Premix Ex Taq II on a CFX96^®^ Real-Time PCR system (Bio Rad, Hercules, CA, USA). The relative expression of different genes was quantified by the comparative Ct method. The ESI† data contains the sequences of the gene-specific primers utilized for qRT-PCR (Table A1). Three biological replicates were performed.

### 4.9. Detection of Antioxidant Enzyme and ROS Signaling Assay

For the measurements of SOD, POD, CAT, and APX activities, previously demonstrated methods were used [49]. These activities (SOD, POD, CAT, APX) were quantified at 560 nm, 470 nm, 240 nm, and 290 nm, at a time range of 60 s, 60 s, 30 s, and 60 s, respectively.

For quantitative analyses, 3 mm diameter leaf disks were immersed in 50 μL distilled water in a 96-well plate and left in the dark overnight. A 100 μL solution comprising 100 μM luminol (Sigma-Aldrich, St. Louis, MO, USA) and 1 μg of horseradish peroxidase was used as a substitute for water. A microplate luminometer was used to measure the luminescence of H_2_O_2_ for 46 min after the addition of ABA (150 μM as described previously) [19].

### 4.10. Statistical Analysis

All experiments were performed with three biological replicates. Data were statistically analyzed to determine the significant differences by Student’s *t*-test and shown as the mean ± SE using TBtools (v2.084) and GraphPad Prism (version 10) [51,52].

## 5. Conclusions

In this study, a genome-wide analysis of *Brassica napus* cultivar ZS11 identified 19 *PSK* genes, which were phylogenetically classified into groups consistent with their homologs in *Arabidopsis thaliana*. These *BnPSK* genes exhibited group-dependent characteristics in chromosomal distribution, conserved motifs, gene structure, and predicted post-translational modification sites, supporting their functional annotation as PSK-related signaling peptides. Expression profiling revealed that a subset of *BnPSK* genes was responsive to drought stress following exogenous ABA treatment, indicating their involvement in ABA-mediated signaling processes. Furthermore, ABA application modulated ROS homeostasis, enhanced antioxidant enzyme activities, and upregulated stress-responsive gene expression, reinforcing the central role of ABA in drought adaptation. In response, we have framed the network analysis around a testable *PSK*–ABA–ROS regulatory module, thereby transforming it from a primarily descriptive network into a hypothesis-driven framework with clear mechanistic implications. Collectively, this study provides a systematic framework linking the *BnPSK* family to ABA-associated stress responses in *B. napus* and lays the foundation for further functional studies to elucidate the precise roles of these peptides in stress resilience.

## Figures and Tables

**Table 1:** Identification and major characteristics of the *BnPSK* gene family.

Gene ID	Name Given	Chr	Start	End	Strand	PSK_Domain	Transmembrane _Region	Signal Peptide	No. of AA	Localization	pI	MW (Da)	GRAVY
*BnaC05G0107300ZS*	*BnPSK1a*	C05	6399434	6399914	+	11-88	9-26	1-27	90	Chloroplast	7.75	9994.5	−0.438
*BnaA06G0087300ZS*	*BnPSK1b*	A06	5183657	5184142	+	11-88	9-26	1-27	90	Chloroplast	6.56	9883.27	−0.523
*BnaA09G0639800ZS*	*BnPSK1c*	A09	61616618	61617671	−	15-87	9-26	-	89	Chloroplast	7.79	9842.38	−0.160
*BnaC08G0497100ZS*	*BnPSK1d*	C08	51403554	51404273	−	12-87	9-26	1-27	89	Chloroplast	6.71	9856.38	−0.238
*BnaC04G0442600ZS*	*BnPSK2a*	C04	56870093	56870515	+	10-83	-	1-22	85	Chloroplast	4.72	9367.78	−0.012
*BnaA04G0150300ZS*	*BnPSK2b*	A04	16302637	16303065	+	10-83	-	1-22	85	Chloroplast	4.71	9459.87	−0.058
*BnaA01G0236600ZS*	*BnPSK3a*	A01	16244641	16245781	+	12-85	5-27	1-22	85	Chloroplast	5.17	9670.19	−0.068
*BnaC01G0307200ZS*	*BnPSK3b*	C01	18058618	18060488	+	11-86	5-27	1-23	86	Chloroplast	5.17	9821.38	−0.098
*BnaC09G0608900ZS*	*BnPSK3c*	C09	67375287	67376119	−	13-98	-	1-25	99	Chloroplast	5.02	11,561.12	−0.737
*BnaA10G0289700ZS*	*BnPSK3d*	A10	25984430	25985269	−	13-98	5-22	1-25	99	Chloroplast	5.03	11,591.13	−0.700
*BnaA09G0466300ZS*	*BnPSK4a*	A09	51621800	51622622	+	10-79	5-24	1-21	79	Chloroplast	6.26	8871.16	−0.235
*BnaC08G0299800ZS*	*BnPSK4b*	C08	38504271	38505119	+	10-79	5-24	1-21	79	Chloroplast	5.05	8888.98	−0.394
*BnaC01G0275000ZS*	*BnPSK4c*	C01	22711566	22712293	−	10-79	5-24	1-21	79	Chloroplast	5.5	8796	−0.228
*BnaA01G0216500ZS*	*BnPSK4d*	A01	13800015	13800738	−	10-79	7-26	1-21	79	Chloroplast	5.17	8712.87	−0.181
*BnaA06G0307600ZS*	*BnPSK5a*	A06	39877634	39877994	+	28-77	1-24	4-23	78	Chloroplast	4.8	8836.02	0.009
*BnaC03G0516300ZS*	*BnPSK5b*	C03	33614473	33615228	−	28-77	7-24	1-24	78	Chloroplast	4.82	8846.06	−0.006
*BnaA02G0415200ZS*	*BnPSK5c*	A02	35638193	35638513	+	29-77	7-24	1-24	77	Chloroplast	4.59	8619.71	−0.065
*BnaC02G0551300ZS*	*BnPSK5d*	C02	12306239	12306929	+	11-77	7-24	1-24	77	Chloroplast	4.59	8629.75	−0.075
*BnaA08G0187700ZS*	*BnPSK6a*	A08	13089801	13090179	−	10-86	-	1-23	87	Chloroplast	4.46	9764.00	−0.309

**Abbreviations:** Chr, Chromosome. AA, amino acids. pI, isoelectric point. MW, molecular weight. GRAVY, grand average of hydropathy.

## Data Availability

The original contributions presented in this study are included in the article/Supplementary Material. Further inquiries can be directed to the corresponding authors.

## Supplementary Materials

The following supporting information can be downloaded at: https://www.mdpi.com/article/10.3390/ijms27021064/s1.

## Abbreviations

The following abbreviations are used in this manuscript:

Asn	Asparagine
ROS	Reactive oxygen species
CREs	*Cis*-acting regulatory elements
Glu	Glutamate
PTM	Post-translational modifications
PSK	Phytosulfokine
POD	Peroxidase
SOD	Superoxide dismutase
ZS11	Zhongshuang11

## Appendix A

**Table A1:** Primers used in this study.

Genes	Forward	Reverse
*BnActin7*	GGAAGCTCCTGGAATCCATGAGA	TCTTTGCTCATACGGTCAGCAATTCC
*BnPSK1a*	ATGATGAAGACTAAGAGTAA	GAGCAGCTACGGTTTTCTTA
*BnPSK1b*	AATATAAAACTCACATACAT	GGAGATGACTTTAAGTCTTG
*BnPSK1c*	GATGAAAACTAGAATGTTG	GTCTTGTGTATAGATGTAAT
*BnPSK1d*	AGAATGTTGATTATCCTCTT	GGAGATATGTTCATGTCTTG
*BnPSK2a*	GGCAAACTTCTCTGCTCTTC	CTGTTTCTGGGTATAGATG
*BnPSK2b*	ATGGCAAACTTCTCTGCTCT	CTGGGTATAGATGTAATCA
*BnPSK3a*	ATGAAGCAAGCCTTGTGCC	ATGCTTATGGTGCTGTGTAT
*BnPSK3b*	GTGCCTCACAGTGTGTGTTC	GGTGCTGTGTATAAATGTAG
*BnPSK3c*	GCTCTTCCTTTTCGTTCTTC	GTGTATGAAGAGAAGGATG
*BnPSK3d*	ATGTCTTACGGTTTATCACC	GAGTGTAGATGTAGTCCAAG
*BnPSK4a*	GGCTAAGTTCACAACCATTT	GGTGTAAATGTAATCGGTGT
*BnPSK4b*	ATGGCCAAGTTCACAACCA	GTGTAAATGTAATCGGTGTG
*BnPSK4c*	GCTCAACGCTAACCTACGC	GTGAGCAACAAGAGTTCG
*BnPSK4d*	GCACAACCATTTTCATCATG	GTCGGTGTGAGCAACAAGAG
*BnPSK5a*	GCGAAGTTCACAGCTTTCT	CTAAAGTTCTTCGTATCAA
*BnPSK5b*	GCCCTCCTCCTCTTCTCCAC	GATTGTGGTTTTGGGTATAG
*BnPSK5c*	CTTCTTTATCATTGTTCTCC	GTGTGTAGATGTAATCAGTA
*BnPSK5d*	CACAACTTTCTTCTTTATC	GGGATTGTGGTTTTGTGTG
*BnPSK6a*	GGAAAATCTCTCTACTTTG	GATATAATCAAGATGAGCCG
*Bn-SOD (Cu-Zn) 2*	GCCTCCTCGTTTCCCCTCCG	TTGTGTCAAAGTAACAACTCC
*Bn-POD*	CCAACAAACTTTCACCACC	TGGTGAGTATATCAGCGCA
*Bn-CAT*	CTTACAGGGTTCGTCCTTCA	GTCATGAGTGACTTCAAAGAA
*Bn-APX*	GGACTTGTTCCCCATCTTA	CTTTGTCATTGAGTCCCATC
*BnDREB2A*	GGGGAGTTAGGCAGAGGATT	CCTCCTTCTTCATCTCTTC
*BnP5CS1*	GGAAGATTGGCTCTTGGTCG	CATTAAGTTGCTTCCTGAAC
*BnLEA1*	CGGTTACGGACGTCGACCT	CCTATTTCGAGCAAATAGTC
*BnNCED3*	CCGTCATTATCATCTTCTCA	CCGGTATTGTCCCGACCACC
*BnbZIP9*	GACAAGAATCTATTATTGAC	GGCAAAAAGAGCGGCTCGCG
