# Supplementary material for: Genome-Wide Identification of PSK Gene Family and Effects of Abscisic Acid (ABA) in Regulating Antioxidant Activity and ROS Signaling Under Drought Stress in Brassica napus
Source: Int J Mol Sci. 2026 Jan 21;27(2):1064. doi: 10.3390/ijms27021064 (PMC12841938; doi:10.3390/ijms27021064)
Supplement: Supplementary file 1 [file ijms-27-01064-s001.zip › ijms-4075585-supplementary.pdf]

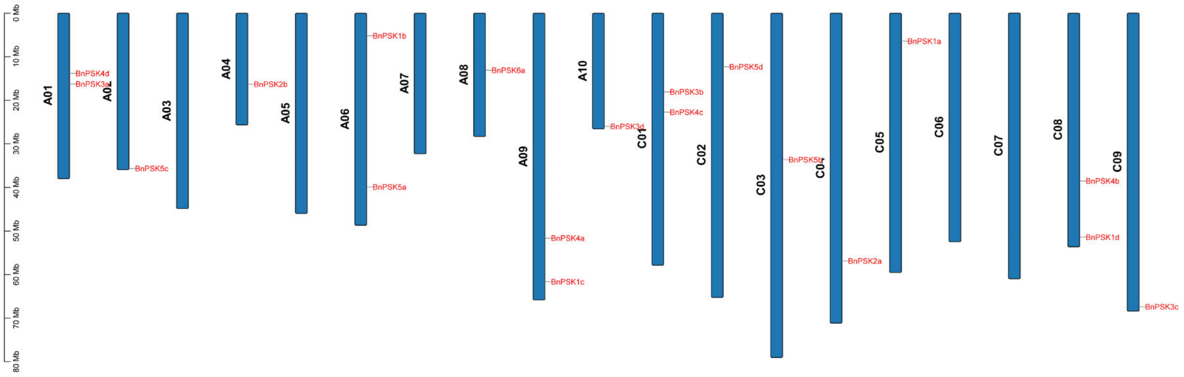

Figure S1. **Chromosomal locations of *BnPSK* genes. Black lines on each chromosome indicating the positions of genes highlighted in red color.**

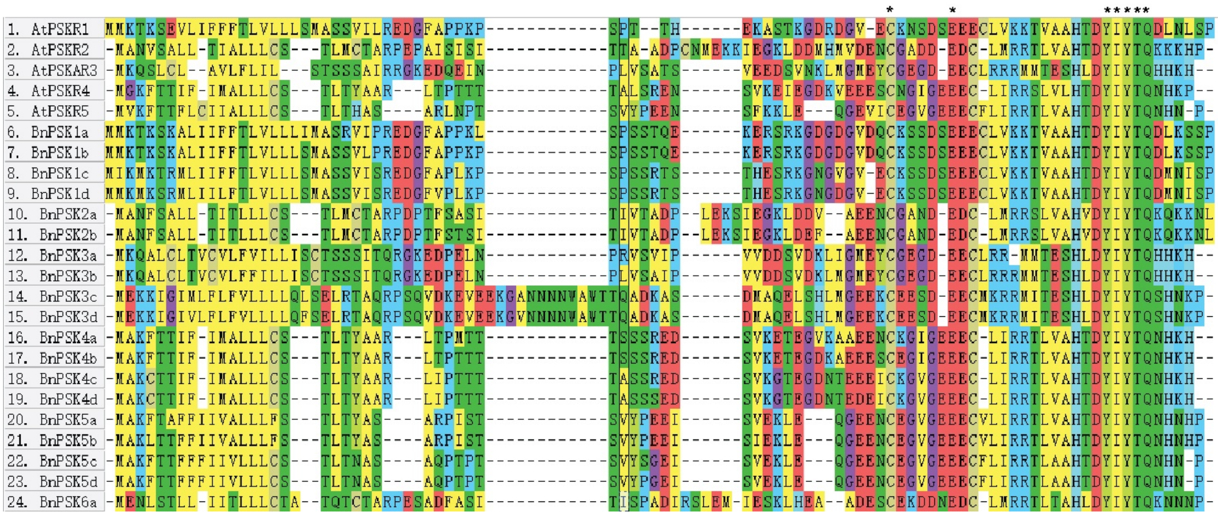

Figure S2. Alignments of the amino acid sequences of PSKs from 19 BnPSKs. Conserved motifs of *B. napus* PSK proteins. Multiple sequence alignment of BnPSK proteins for conserved motifs. Alignment was performed using ClustalW and presented by GeneDoc software. Residues with '*' showed 100% conservativeness.

Motif Logos | **Motif Sequences**

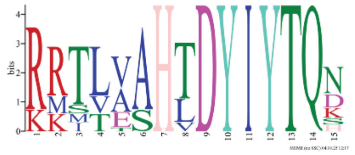

RRTLVAHTDYIYTQB

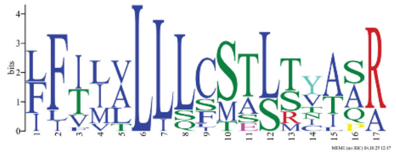

LFILVLLLCSTLTYAAR

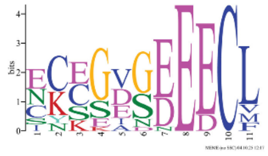

ECEGVGEEECL

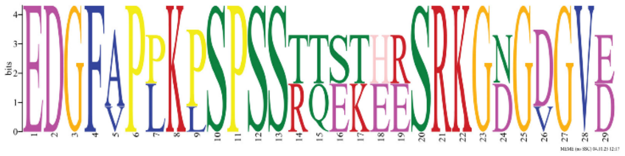

EDGFAPPKPSPSSRTEKEESRKGDGDGVD

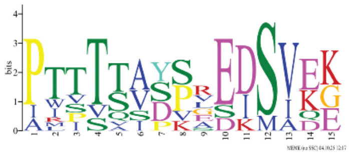

PTTTTADPREDSVEK

Figure S3. Motif logos obtained from the weblogo webserver. BnPSK-specific motifs logos showing the amino acids were generated using Web logo3 and the bit score for each position in the sequence are indicated to the left.
